# Supplementary material for: Factors associated with unskilled birth attendance among women in sub-Saharan Africa: A multivariate-geospatial analysis of demographic and health surveys
Source: PLoS One. 2023 Feb 2;18(2):e0280992. doi: 10.1371/journal.pone.0280992 (PMC9894461; doi:10.1371/journal.pone.0280992)
Supplement: Attachment — Submitted filename: PONE-D-21-36273.pdf [file pone.0280992.s002.pdf]

PLOS ONE

Factors associated with unskilled birth attendance among women in Sub-Saharan Africa: a multilevel geospatial analysis of demographic and health surveys

--Manuscript Draft--

**Manuscript Number:**	PONE-D-21-36273
**Article Type:**	Research Article
**Full Title:**	Factors associated with unskilled birth attendance among women in Sub-Saharan Africa: a multilevel geospatial analysis of demographic and health surveys
**Short Title:**	Factors associated with unskilled birth attendance among women in Sub-Saharan Africa
**Corresponding Author:**	Kwamena Sekyi Dickson, PhD<br>University of Cape Coast<br>Cape Coast, GHANA
**Keywords:**	unskilled birth attendants; traditional birth attendants; Sub-Saharan Africa (SSA); Demographic and Health Surveys (DHS); geospatial analysis; Social demography; Public health
**Abstract:**	Background: Studies have indicated that unskilled birth attendance is associated with maternal morbidity, disability, and death. In many Sub-Saharan African (SSA) countries, it is estimated that more than 50% of pregnant women utilise the services of unskilled birth attendants which partly explains why the region ranks as the highest in terms of maternal and neonatal deaths. Despite this, little evidence exists on prevailing geospatial variations and the factors underscoring the patterns of unskilled birth attendance in the region. This study analysed the geospatial disparities and factors associated with unskilled birth attendance in SSA.<br>Methods: The study is based on data from 30 SSA countries captured in the most recent (2010-2019) demographic and health surveys (DHS). A total of 200,736 women aged between 15-49 years were included in the study. Geospatial methods including spatial autocorrelation and hot spot analysis as well as logistic regression models were used to analyse the data.<br>Results: There were random spatial variations in unskilled birth attendance in SSA, with the hotspot in Chad, whereas South Africa and the Democratic Republic of Congo showed coldspots. Urban residence, rich wealth status, higher education, older current age and age at birth, cohabitation, lower birth order, not desiring birth, not having money problems for healthcare were associated with reduced odds of unskilled birth attendance. Higher odds of unskilled birth attendance were associated with teenagehood, no education, widowhood/divorce, unemployment, lack of media exposure, big problem with distance to a health facility, less than four antenatal care visits, and demise of an under-five child as well as virtually all the countries compared to Burundi.<br>Conclusion: Random geospatial disparities in unskilled birth attendance exist in SSA, coupled with various associated socio-demographic determinants. Specific geospatial hot spots of unskilled birth attendance in SSA can be targeted for specialised interventions to alleviate the prevailing disparities.
**Order of Authors:**	Isaac Yeboah Addo, PhD
	Evelyn Acquah
	Samuel H. Nyarko
	Ebenezer N.K. Boateng
	Kwamena Sekyi Dickson, PhD
**Additional Information:**	
**Question**	**Response**
**Financial Disclosure**	The author(s) received no specific funding for this work

Powered by Editorial Manager® and ProduXion Manager® from Aries Systems Corporation

Enter a financial disclosure statement that describes the sources of funding for the work included in this submission. Review the submission guidelines for detailed requirements. View published research articles from *PLOS ONE* for specific examples.<br><br>This statement is required for submission and **will appear in the published article** if the submission is accepted. Please make sure it is accurate.<br><br>**Unfunded studies**<br>Enter: *The author(s) received no specific funding for this work.*<br><br>**Funded studies**<br>Enter a statement with the following details:<br>• Initials of the authors who received each award<br>• Grant numbers awarded to each author<br>• The full name of each funder<br>• URL of each funder website<br>• Did the sponsors or funders play any role in the study design, data collection and analysis, decision to publish, or preparation of the manuscript?<br>• **NO** - Include this sentence at the end of your statement: *The funders had no role in study design, data collection and analysis, decision to publish, or preparation of the manuscript.*<br>• **YES** - Specify the role(s) played.<br><br>* typeset	
**Competing Interests**<br><br>Use the instructions below to enter a competing interest statement for this submission. On behalf of all authors, disclose any competing interests that could be perceived to bias this work—acknowledging all financial support and any other relevant financial or non-financial competing interests.<br><br>This statement is required for submission and **will appear in the published article** if the submission is accepted. Please make	The authors have declared that no competing interests exist

sure it is accurate and that any funding sources listed in your Funding Information later in the submission form are also declared in your Financial Disclosure statement.<br><br>View published research articles from *PLOS ONE* for specific examples.<br><br>**NO authors have competing interests**<br><br>Enter: *The authors have declared that no competing interests exist.*<br><br>**Authors with competing interests**<br><br>Enter competing interest details beginning with this statement:<br><br>*I have read the journal's policy and the authors of this manuscript have the following competing interests: [insert competing interests here]*<br><br>* typeset	
**Ethics Statement**<br><br>Enter an ethics statement for this submission. This statement is required if the study involved:<br><br>• Human participants<br>• Human specimens or tissue<br>• Vertebrate animals or cephalopods<br>• Vertebrate embryos or tissues<br>• Field research<br><br>Write "N/A" if the submission does not require an ethics statement.<br><br>General guidance is provided below. Consult the submission guidelines for detailed instructions. **Make sure that all information entered here is included in the Methods section of the manuscript.**	N/A

Powered by Editorial Manager® and ProduXion Manager® from Aries Systems Corporation

**Format for specific study types**<br><br>**Human Subject Research (involving human participants and/or tissue)**<br>• Give the name of the institutional review board or ethics committee that approved the study<br>• Include the approval number and/or a statement indicating approval of this research<br>• Indicate the form of consent obtained (written/oral) or the reason that consent was not obtained (e.g. the data were analyzed anonymously)<br><br>**Animal Research (involving vertebrate animals, embryos or tissues)**<br>• Provide the name of the Institutional Animal Care and Use Committee (IACUC) or other relevant ethics board that reviewed the study protocol, and indicate whether they approved this research or granted a formal waiver of ethical approval<br>• Include an approval number if one was obtained<br>• If the study involved *non-human primates*, add *additional details* about animal welfare and steps taken to ameliorate suffering<br>• If anesthesia, euthanasia, or any kind of animal sacrifice is part of the study, include briefly which substances and/or methods were applied<br><br>**Field Research**<br><br>Include the following details if this study involves the collection of plant, animal, or other materials from a natural setting:<br>• Field permit number<br>• Name of the institution or relevant body that granted permission	
**Data Availability**<br><br>Authors are required to make all data underlying the findings described fully available, without restriction, and from the time of publication. PLOS allows rare exceptions to address legal and ethical concerns. See the PLOS Data Policy and FAQ for detailed information.	Yes - all data are fully available without restriction

A Data Availability Statement describing where the data can be found is required at submission. Your answers to this question constitute the Data Availability Statement and **will be published in the article**, if accepted.<br><br>**Important:** Stating 'data available on request from the author' is not sufficient. If your data are only available upon request, select 'No' for the first question and explain your exceptional situation in the text box.<br><br>Do the authors confirm that all data underlying the findings described in their manuscript are fully available without restriction?	
**Describe where the data may be found in full sentences. If you are copying our sample text, replace any instances of XXX with the appropriate details.**<br><br>• If the data are **held or will be held in a public repository**, include URLs, accession numbers or DOIs. If this information will only be available after acceptance, indicate this by ticking the box below. For example: *All XXX files are available from the XXX database (accession number(s) XXX, XXX.).*<br>• If the data are all contained **within the manuscript and/or Supporting Information files**, enter the following: *All relevant data are within the manuscript and its Supporting Information files.*<br>• If neither of these applies but you are able to provide **details of access elsewhere**, with or without limitations, please do so. For example:<br><br>*Data cannot be shared publicly because of [XXX]. Data are available from the XXX Institutional Data Access / Ethics Committee (contact via XXX) for researchers who meet the criteria for access to confidential data.*<br><br>*The data underlying the results presented in the study are available from (include the name of the third party*	https://dhsprogram.com/data/available-datasets.cfm

and contact information or URL).<br>• This text is appropriate if the data are owned by a third party and authors do not have permission to share the data.<br><br>* typeset	
Additional data availability information:	

Manuscript

Click here to access/download;Manuscript;FULL MANUSCRIPT UBA.docx

Factors associated with unskilled birth attendance among women in Sub-Saharan Africa: a multilevel geospatial analysis of demographic and health surveys

Isaac Yeboah Addo [1], Evelyn Acquah [2], Samuel H. Nyarko [3], Ebenezer N.K. Boateng [4], Kwamena Sekyi Dickson [5]

[1]Centre for Social Research in Health; UNSW Sydney, 2052, Australia; Email: i.addo@unsw.edu.au

[2] Centre for Health Policy and Implementation Research, Institute of Health Research, University of Health and Allied Sciences, Ghana; Email: eacquah@uhas.edu.gh

[3]Department of Epidemiology, Human Genetics, and Environmental Sciences, University of Texas Health Science Center at Houston, USA; Email: samharrenson@gmail.com

[4]Department of Geography and Regional Planning, University of Cape Coast, Ghana; Email: ebenezer.boateng@stu.ucc.edu.gh

[5]Department of Population and Health, University of Cape Coast, Ghana; Email: nadicx@gmail.com

Short Title: Unskilled Birth Attendance among Women in Sub-Saharan Africa

Corresponding authors: Kwamena Sekyi Dickson, Email: nadicx@gmail.com and Isaac Yeboah Addo, Email: i.addo@unsw.edu.au

Abstract

Background: Studies have indicated that unskilled birth attendance is associated with maternal morbidity, disability, and death. In many Sub-Saharan African (SSA) countries, it is estimated that more than 50% of pregnant women utilise the services of unskilled birth attendants which partly explains why the region ranks as the highest in terms of maternal and neonatal deaths. Despite this, little evidence exists on prevailing geospatial variations and the factors underscoring the patterns of unskilled birth attendance in the region. This study analysed the geospatial disparities and factors associated with unskilled birth attendance in SSA.

Methods: The study is based on data from 30 SSA countries captured in the most recent (2010-2019) demographic and health surveys (DHS). A total of 200,736 women aged between 15-49 years were included in the study. Geospatial methods including spatial autocorrelation and hot spot analysis as well as logistic regression models were used to analyse the data.

Results: There were random spatial variations in unskilled birth attendance in SSA, with the hotspot in Chad, whereas South Africa and the Democratic Republic of Congo showed coldspots. Urban residence, rich wealth status, higher education, older current age and age at birth, cohabitation, lower birth order, not desiring birth, not having money problems for healthcare were associated with reduced odds of unskilled birth attendance. Higher odds of unskilled birth attendance were associated with teenagehood, no education, widowhood/divorce, unemployment, lack of media exposure, big problem with distance to a health facility, less than four antenatal care visits, and demise of an under-five child as well as virtually all the countries compared to Burundi.

Conclusion: Random geospatial disparities in unskilled birth attendance exist in SSA, coupled with various associated socio-demographic determinants. Specific geospatial hot spots of unskilled

birth attendance in SSA can be targeted for specialised interventions to alleviate the prevailing disparities.

Keywords: unskilled birth attendants; traditional birth attendants; Sub-Saharan Africa (SSA); Demographic and Health Surveys (DHS); geospatial analysis; logistic regression models

Introduction

Historically, traditional or unskilled birth attendants defined as “persons who assist a mother during childbirth and who initially acquired their skills by delivering babies themselves or through apprenticeship to other traditional birth attendants” (1), have been the primary caregivers for women during pregnancy and childbirth in Sub-Saharan Africa (SSA) (2). In addition to their provision of care during pregnancy and child delivery, unskilled birth attendants play other roles, such as providing family planning information and advising pregnant women about their nutritional requirements (3). Due to challenges associated with collecting maternal health data in some SSA countries (1), an aggregated estimate of unskilled birth attendance in the region is limited. However, current anecdotal reports suggest that more than one in two pregnant women (>50%) utilise the services of unskilled birth attendants in many SSA countries (4).

Employing the services of unskilled birth attendants in the management of pregnancy and child delivery is known to be associated with a high risk of maternal and natal morbidity, disability, and even death (5). Unskilled birth attendants usually lack the required knowledge, skills, or resources to balance risk with benefits during pregnancy and child delivery, and may have difficulty managing common pregnancy or childbirth complications, such as hemorrhage, eclampsia, and obstructed labour (5). It is therefore believed that utilisation of services from unskilled birth attendants during pregnancy and childbirth plays a significant contribution to the level of maternal and natal mortality in SSA which is ranked as the highest in the world- more than two-thirds of maternal deaths per annum or 68% or 533 maternal deaths per 100,000 live births, or 200,000 maternal deaths (6).

Given the assumption that many women in SSA continue to utilise the services of unskilled birth attendants rather than professional or skilled birth attendants, it is important to

understand the factors accounting for these utilisations. Understanding these factors is essential for developing context-specific interventions tailored toward more specific population characteristics which in turn can inform efforts to reduce maternal deaths. However, to date, no empirical study, to the best of our knowledge, has examined the utilisation of unskilled birth attendants in the whole of SSA. The available studies on unskilled birth attendants were focused on only single countries in Africa, e.g., Olakunde, Adeyinka (5) for Nigeria, or explored perceptions of women about unskilled birth attendants from a qualitative perspective, e.g.,-Gurara, Muyldermans (7) for Ethiopia and Selepe and Thomas (8) for South Africa (7-12).

Previous studies focused on skilled birth attendance among women in SSA have suggested that availability of a healthcare facility, distance between home and the healthcare facility, socio-economic status of pregnant women, place of residence, birth parity, ethnicity, previous contact with a healthcare system, and levels of education are associated with the use of services from skilled birth attendants during pregnancy and delivery (5, 13-16). However, a significant gap in current knowledge is the extent to which these factors apply to women who utilise the services of unskilled birth attendants. Exploring women's use of services from unskilled birth attendants and the factors associated with the use of such services will provide important supporting evidence for the development and implementation of evidence-based interventions. Using representative Demographic and Health Survey (DHS) data for women aged 15-49 years from 30 SSA countries, this study examined factors associated with unskilled birth attendance among women in SSA. The study further explored the levels of service use from unskilled birth attendants among women in SSA as well as the hot and cold spot countries associated with the use of such services. Exploring these factors can be useful to maternal health

promoters, health practitioners, policymakers, and global health advocates addressing the unacceptable level of maternal mortality in SSA.

Materials and Methods

Data source

The study analysed data from a pool of the most recent demographic and health surveys (DHS) of 30 countries in Sub-Saharan Africa (SSA), conducted between 2010 and 2019. The surveys comprised nationally representative samples of women in their reproductive age groups (15-49 years), selected based on a two-stage stratified cluster sampling procedure. The DHS generates reliable data on fertility, family planning, infant and child mortality, maternal and child health, among others, and is suitable for this study. Both standard and continuous DHS data were obtained for countries such as Angola (AO), Burkina Faso (BF), Burundi (BD), Cameroon (CM), Chad (TD), Comoros (KM), Congo (CG), Cote D'Ivoire (CI), Democratic Republic of Congo (CD), Ethiopia (ET), Gabon (GA), Gambia (GM), Ghana (GH), Guinea (GN), Kenya (KE), Lesotho (LS), Liberia (LB), Malawi (MW), Mali (ML), Mozambique (MZ), Namibia (NM), Nigeria (NG), Rwanda (RW), Sierra Leone (SL), South Africa (ZA), Tanzania (TZ), Togo (TG), Uganda (UG), Zambia (ZM), and Zimbabwe (ZW). The unit of analysis comprised 200,736 women aged from 15 to 49 years selected from various households in these countries. The sample was limited to the last births of women within the five years preceding the surveys.

Study variables and measurements

The outcome of interest was unskilled birth attendance. This was based on the person who assisted during the delivery of the last child. This comprised multiple categories of persons such

as a doctor, nurse/midwife, community health officer/nurse, traditional birth attendant, traditional health volunteer, community/village health volunteer, relatives, and others. In this study, we created a binary outcome where deliveries assisted by traditional birth attendants, traditional health volunteers, community/village health volunteers, relatives, and others were measured as unskilled birth attendance while all others were considered skilled birth attendance (13, 17).

Also, various explanatory variables, of which most have been established by previous studies relating to the study outcome (17-19), were considered in the study. These include age (Less than 20 years, 20 – 29 years, and 30 – 49 years), level of education (No education, Primary, Secondary, Higher), wealth status (Poorest, Poorer, Middle, Richer, Richest), marital status (Never in a union, Married, Cohabitation, Widowed, Divorced, Separated), occupation (Not working, Working), age at first birth (Less than 20 years, 20 – 29 years, 30 – 49 years), birth order (1, 2 – 3, 4 or more), mass media exposure (No, Yes), getting medical help for self: permission to go (Big problem, Not a big problem), getting medical help for self: getting the money needed for treatment (Big problem, Not a big problem), getting medical help for self: distance to health facility (Big problem, Not a big problem), desire for birth (Then, Later, No more), antenatal care visits (Less than 4, 4 or more), under five mortality (No, Yes), place of residence (Urban, Rural) and country.

Analytical procedure

We initially estimated the proportion of unskilled birth attendance by country and the socio-demographic characteristics of the respondents. Next, multivariable analysis was performed using a logistic regression model to estimate associations between unskilled birth attendance and the selected socio-demographic characteristics of the respondents. Multicollinearity test was

performed for each variable showing a mean-variance inflation factor (VIF) of 1.61 for the variables in the models. Unadjusted (Model 1) and adjusted odds ratios (Model 2) were then calculated for each variable at a 95 % confidence interval. Stata (Version 14) was used to process and analyse the data. The results were sample-weighted to address any over-sampling and under-sampling in the total sample.

Geospatial analysis

With regards to the geospatial analysis, the proportions data on unskilled birth attendance were merged with the shapefiles for each country using ArcMap version 10.5. Spatial autocorrelation (Global Moran's I) tool was used to explore the distribution of unskilled birth attendance in SSA after a hypothesis was set:

H_0: there is a random distribution of unskilled birth attendance in SSA

H_1: the distribution of unskilled birth attendance in SSA is not random

This spatial autocorrelation (Global Moran's I) tool was used to assess whether the distribution of unskilled birth attendance in SSA is random, clustered, or dispersed across the selected countries. The results to determine the type of distribution is dependent on the Moran's I index and the z-score or p-value. A positive and negative Moran's I index value implies a likelihood of clustering and dispersion respectively. The z-score or p-value indicates statistical significance, and the null hypothesis is accepted if the z-score is -1.65-1.65 and if the p-value is not statistically significant. In addition, a hotspot analysis (Getis-Ord G) was conducted to identify areas with relatively higher (hotspot) and lower (coldspot) occurrence of unskilled birth attendance amongst the selected countries based on z-score and p-value. A higher z-score and lower p-value show a higher probability of a phenomenon occurring at a place. The Anselin

Local Moran's I cluster, and outlier analysis was also conducted to ascertain statistically significant spatial outliers. This output showed outliers in the distribution using permutations such as high-high, low-low, high-low, low-high, and not significant.

Results

Unskilled birth attendance by country

Table 1 shows the number of included women by country and the proportion of unskilled birth attendance for the most recent surveys (2010 to 2019). Of the 200,736 women included in the study, the majority (8%) were from Burundi and the minority (1%) were from Lesotho. Chad had the highest proportion (69%) of women who utilised the services of unskilled birth attendants whereas the least proportion (3%) were from South Africa. Overall, about one in four women (27%) in SSA utilised the services of an unskilled birth attendant during pregnancy or delivery.

[Please Insert Table 1 here]

Socio-demographic characteristics of women and unskilled birth attendance

Four in ten women with no education, poorest wealth status, and those who had less than four antenatal visits utilised the services of an unskilled birth attendant (Table 2). More than a quarter of the women with no exposure to mass media messages (36.7%), with four or more children (34.3%), with permission (35.7%), and who regarded distance to a health facility as a big problem (36.5%) utilised the services of an unskilled birth attendant (Table 2). Three in ten women who reported that they had experienced the death of their under-five child utilised the services of an unskilled birth attendant (Table 2).

[Please Insert Table 2 here]

Factors associated with unskilled birth attendance

Table 3 displays the results for all the logistic regression models presented in crude odds ratios (cOR) and adjusted odds ratios (aOR) with their respective 95% confidence intervals (Cis). The findings show that place of residence, wealth status, age, level of education, marital status, occupation, age at first birth, birth order, mass media exposure, permission, getting the money needed for care, distance to a health facility, desire for birth, antenatal care visits, experience of death of an under-five child, and country of residence had a significant relationship with unskilled birth attendance.

A lesser likelihood of utilising the services of unskilled birth attendants was observed among urban women (AOR= 0.53, CI=0.51, 0.55) compared to rural women. Those with the richest wealth status were less likely (AOR=0.23, CI=0.22, 0.24) to utilise the services of unskilled birth attendants compared to those with the poorest wealth status (Table 3). Women who had no more desire to have children (OR=0.86, CI=0.83,0.89), those who did not have a big problem getting money needed for care (OR=0.95, CI=0.92, 0,98) had a lesser likelihood of utilising the services of unskilled birth attendants compared to women who then desired to have more children and those who had a big problem getting money needed for care (Table 3).

Our findings also showed that teenagers (less than 20 years) had a higher likelihood (AOR=1.11, CI=1.05, 1.17) of utilising the services of unskilled birth attendants during delivery compared to women aged 20 – 29 years. Women with no education had a higher odd (AOR=1.57, CI=1.52, 1.62) of utilising the services of unskilled birth attendants during delivery

compared with those with primary education. Women who were not exposed to mass media had a higher likelihood (AOR=1.25, CI=1.22, 1.29) of utilising the services of unskilled birth attendants during delivery compared to those who were exposed to mass media (see Table 3).

Furthermore, the women who saw distance to a healthcare facility as a big problem had a higher likelihood (AOR=1.49, CI=1.45, 1.53) of utilising the services of unskilled birth attendants during delivery compared to women who stated that distance to healthcare facilities was not a big problem. Women who had less than 4 antenatal care visits had a higher odd (AOR=1.10, CI=1.04, 1.17) of utilising the services of unskilled birth attendants during delivery compared to women who had 4 or more antenatal care visits. Women who had experienced the demise of their under-five child were more likely (AOR=1.10, CI=1.04, 1.17) to utilise the services of unskilled birth attendants during delivery compared to those who had not experienced the death of their under-five child.

[Please Insert Table 3 here]

Spatial distribution

Results from the spatial autocorrelation analysis (Figure 1) revealed that the spatial variations in unskilled birth attendance in Sub-Saharan Africa (SSA) were random. Therefore, the alternate hypothesis which states that the spatial distribution of unskilled birth attendance is not randomised was not accepted. Thus, the occurrence of unskilled birth attendance in SSA is not by chance.

[Please Insert Figure 1 here]

Although the occurrence of unskilled birth attendance was found to be random, the Hotspot Analysis (Figure 2) showed that at a 90% confidence level, the occurrence of unskilled birth attendance was likely to be found in Chad compared to the other selected countries. This implies a higher tendency for women in Chad to deliver without a skilled birth attendant than the other selected countries. Also, two countries were found to have coldspots at a 90% confidence level (Figure 2). These countries were South Africa and the Democratic Republic of Congo. Concerning all the selected countries for this study, the occurrence of unskilled birth attendance in South Africa and the Democratic Republic of Congo was found to be less likely.

[Please Insert Figure 2 here]

Results from the cluster and outlier analysis (Figure 3) showed no high-high, high-low and low-high clusters. The cluster and outlier analysis showed that the Democratic Republic of Congo and Lesotho had low UBA and were surrounded by countries with low UBA. This is evident from the hotspot analysis presented in Figure 3.

[Please Insert Figure 3 here]

Discussion

Surveillance data from relevant international bodies, such as the World Health Organisation (WHO) and the United Nations Children's Fund (UNICEF), have consistently indicated that there is a gross geospatial disparity in the incidence of maternal mortality between low- and

high-income countries (4, 6, 20, 21), with Sub-Saharan Africa (SSA) ranking as the region with the highest incidence of maternal deaths. Evidence further suggests that unskilled birth attendance contributes significantly to maternal health complications and deaths in SSA (21). This study, therefore, examined levels of unskilled birth attendance among women in SSA and the factors associated with the utilisation of such services. The study further explored the hot and cold spot countries related to the use of services from unskilled birth attendants in SSA.

In general, the findings indicate significant variations in unskilled birth attendance among women in SSA, and these variations are strongly associated with differences in their socio-demographic and economic characteristics. Specifically, women in rural areas, with low income, aged below 20 years, with no or low levels of education, with low exposure to mass media information, located far away from healthcare facilities, and with low levels of antennal attendance (<4 hospital or clinic attendance) were likely to utilise pregnancy and birth-related services from unskilled birth attendants. These findings are consistent with a number of country-specific studies in SSA, such as the works of Namusonge, Kabue (22) for Kenya, and Bediako, Boateng (17) for Ghana. Our findings also show a reflection of outcomes from previous studies focused on skilled birth attendance among women in SSA (5, 13-16). The gross disparities in unskilled birth attendance among the various characteristics of women in this present study suggest that socio-economic inequalities among women are strong determinants of their health. Many SSA countries have fragile health care systems with a lack of skilled birth attendants and aggravated by an increased cost of medical care, geographic inaccessibility of health care facilities, among others (23). Hence, many socially disadvantaged women, such as those with no or low exposure to media advocacies on maternal health, low levels of education, low incomes,

and living in rural areas, are likely to continue using the relatively cheaper and easily accessible services of unskilled birth attendants.

It can be logically assumed that women who had witnessed the death of a child would seek the services of skilled or professional birth attendants in their subsequent pregnancies and child deliveries. Interestingly, our findings indicate that women who had ever experienced the demise of an under-five child were likely to continue using services from unskilled birth attendants. This result is difficult to explain. However, consistent with the findings of Imogie (10) and Jossy van den Boogaard and Jelle (14), this may imply that many women had no alternatives to services from unskilled birth attendants due to factors such as long distance to healthcare facilities. Another possible explanation in line with the inferential work of Imogie (10) is that many women did not attribute the demise of their under-five children to the services of unskilled birth attendants. In a qualitative study in Ghana (24), for instance, women ranked services of unskilled birth attendants higher than skilled birth attendants and noted that traditional birth attendants are more experienced and understanding of their psychosocial needs than young professional midwives. These findings indicate the need to amplify education for women in SSA on the benefits of seeking professional care and also suggest a call for improvements in the psychosocial interactions between healthcare professionals and women in SSA.

The outcomes from our geospatial analysis further suggest significant spatial variations in unskilled birth attendance among women in the 30 SSA countries, but those variations did not occur by chance. This means that although the use of services from unskilled birth attendants during pregnancy and delivery in SSA is a holistic problem, the situation varies by country of residence. Notably, with an outstanding level of unskilled birth attendance (69%), Chad can be

classified as a hotspot and high-risk country for unskilled birth attendance compared to its neighbouring countries. On the other hand, South Africa (3%) can also be classified as a coldspot country where women predominantly seek maternal health care from skilled birth attendants. The government of Chad and concerned health care institutions in the country need to seriously consider developing urgent interventions to address the high rate of unskilled birth attendance among women in the country.

Strengths and limitations

This study contains novel findings on factors associated with unskilled birth attendance in Sub-Saharan Africa (SSA). To the best of our knowledge, this is the first study on this topic that has employed nationally representative samples drawn from 30 SSA countries. We used a large dataset comprising 200,736 women aged between 15-49 years which makes our results compelling. Findings from this study are also based on rigorous logistic regression and supplemented by spatial autocorrelation analyses which are unprecedented in the context of this topic. Despite these strengths, the study had some notable shortcomings. Firstly, the cross-sectional nature of the study design limits causal inferences between the participant characteristics and unskilled birth attendance. Secondly, there is a possibility of social desirability and data recall bias in the responses as most questions were answered through self-reporting method. For instance, women from cultural orientations where utilisation of services from unskilled birth attendants is stigmatised are likely to provide favourable responses to please the ethos of their cultural orientations. Some women may also regard unskilled birth attendance as skilled or professional care leading to either underestimation or overestimation of the reported proportions. Additionally, only women were included in this study meaning that the opinions of

partners are limited in the findings. Lastly, we assumed that variables, such as cultural norms and attitudes of health service providers would have been interesting to explore in the context of this study, but such variables were not captured in the DHS dataset.

Implications for policy and practice

The multiple factors associated with unskilled birth attendance among the women in this study imply that there is a need for multi-faceted and local-specific policies and programmes in addressing the problem of unskilled birth attendance in SSA. Thus, a “one size fits all” approach may not be efficient in addressing unskilled birth attendance among women in the region. Some possibly useful strategies may include the provision of routine localised or rural health education for pregnant women about the importance of utilising the services of skilled birth attendants. Governments in SSA need to also make it a priority to extend healthcare facilities to rural areas to bridge the distance gap and improve the accessibility of skilled birth services for pregnant women in disadvantaged settings.

It is also important to recognise that although there is a general advocate for skilled birth attendance, there are some situations where service use from unskilled birth attendants may be non-negotiable. For instance, access to healthcare can be challenging in many conflict-affected countries or areas in protracted armed conflicts where healthcare workers have vacated their posts due to fear of persecution (3). Some regions in SSA also have significant scarcities in skilled birth attendants making access to professional care limited (9). As revealed in this study, long-distance to healthcare facilities, rural residency, and low-income levels can also deter women from accessing professional care during pregnancy and child delivery. In such circumstances, health policy advocates, local governments, and concerned healthcare institutions

can collaborate to develop a system whereby unskilled birth attendants are well trained and equipped with the needed modern equipment to facilitate optimal care for pregnant women. In recent times where telehealth and telemedicinal approaches are emerging as useful resources, the role played by unskilled birth attendants can be integrated into local healthcare systems and adequately monitored. A practical example is a situation in Timor-Leste where unskilled birth attendants were incorporated into a family health promoter programme which played crucial roles in delivering and increasing access to reproductive health services in rural communities of the nation. Although it required long-term dedication and effective collaborations, the current reduction in maternal mortality ratio in Timor-Leste is encouraging and serves to illustrate how such approaches can be useful. (25).

Conclusions

This study has confirmed that many women in Sub-Saharan Africa (SSA) continue to utilise the services of unskilled birth attendants during pregnancy and delivery. The study also concludes that women in rural areas, with low income, aged below 20 years old, with no or low levels of education, less exposed to mass media information, distanced away from healthcare facilities, and with low levels of antennal attendance (<4 hospital or clinic attendance) are likely to utilise the services of unskilled birth attendants. These findings suggest that multiple interventions focused on these contextual factors are needed in SSA. We propose an integrated approach where skilled birth attendants are prioritised, but in situations where there are shortages of skilled birth attendants, unskilled birth attendants can be trained, licensed, and monitored to save the lives of pregnant women. Extending maternal education and healthcare facilities or centres to rural areas should be a priority for managers of healthcare systems in SSA. Intensifying

education for pregnant women about the importance of using skilled or professional healthcare during pregnancy, delivery, and post-delivery periods is also needed. Future studies can enhance a deeper understanding of these results by exploring our findings from qualitative perspectives.

References

1. World Health O. Traditional birth attendants: a joint WHO/UNFPA/UNICEF statement: World Health Organization; 1992.

2. Aziato L, Omenyo CN. Initiation of traditional birth attendants and their traditional and spiritual practices during pregnancy and childbirth in Ghana. BMC Pregnancy and Childbirth. 2018;18(1):64.

3. Chi PC, Urdal H. The evolving role of traditional birth attendants in maternal health in post-conflict Africa: A qualitative study of Burundi and northern Uganda. SAGE Open Medicine. 2018;6:2050312117753631.

4. World Health Organization. Skilled birth attendants Geneva, Switzerland: WHO; 2021 [Cited on 27 September 2021]. Available from: https://www.who.int/reproductivehealth/topics/mdgs/skilled_birth_attendant/en/.

5. Olakunde BO, Adeyinka DA, Mavegam BO, Olakunde OA, Yahaya HB, Ajiboye OA, et al. Factors associated with skilled attendants at birth among married adolescent girls in Nigeria: evidence from the Multiple Indicator Cluster Survey, 2016/2017. International Health. 2019;11(6):545-50.

6. United Nations Children's Fund (UNICEF). Maternal mortality United States: UNICEF; 2019 [Available from: https://data.unicef.org/topic/maternal-health/maternal-mortality/#:~:text=Sub%2DSaharan%20Africans%20suffer%20from,maternal%20deaths%20per%20year%20worldwide.

7. Gurara M, Muyldermans K, Jacquemyn Y, Van geertruyden J-P, Draulans V. Traditional birth attendants’ roles and homebirth choices in Ethiopia: A qualitative study. Women and Birth. 2020;33(5):e464-e72.

8. Selepe HL, Thomas DJ. The beliefs and practices of traditional birth attendants in the Manxili area of KwaZulu, South Africa: a qualitative study. J Transcult Nurs. 2000;11(2):96-101.

9. Hodnett E. Traditional birth attendants are an effective resource. BMJ. 2012;344:e365.

10. Imogie AO. The Practice Of Traditional Birth Attendants And Women’s Health In Nigeria Australia: The Regional Institute; 2021 [Available from: http://www.regional.org.au/au/mwia/papers/full/28_imogie.htm.

11. Ngunyulu RN, Mulaudzi FM, Peu MD. Traditional birth attendants’ experiences during the provision of post-natal care in Mopani District, Limpopo province of South Africa. Health SA Gesondheid; Vol 25 (2020)DO - 104102/hsagv25i01468. 2020;25:1468.

12. Nolte A. Traditional birth attendants in South Africa: professional midwives' beliefs and myths. Curationis. 1998;21(3):59-66.

13. Dickson KS, Adde KS, Ameyaw EK. Women empowerment and skilled birth attendance in sub-Saharan Africa: A multi-country analysis. PLOS ONE. 2021;16(7):e0254281.

14. Jossy van den Boogaard BAJCMLAM, Jelle S. Skilled or Traditional Birth Attendant? Choices of Communities in Lukulu District, Rural Zambia. World Health & Population. 2008;10(1):34-43.

15. Kyeyune H. Pregnant women favor traditional birth attendants in Uganda despite ban Uganda: Anadolu Agency; 2020 [Available from: https://www.aa.com.tr/en/africa/pregnant-women-favor-traditional-birth-attendants-in-uganda-despite-ban/1750631.

16. Mekonnen T, Dune T, Perz J, Ogbo FA. Trends and predictors of the use of unskilled birth attendants among Ethiopian mothers from 2000 to 2016. Sexual & Reproductive Healthcare. 2021;28:100594.

17. Bediako VB, Boateng ENK, Owusu BA, Dickson KS. Multilevel geospatial analysis of factors associated with unskilled birth attendance in Ghana. PLOS ONE. 2021;16(6):e0253603.

18. Doctor HV, Dahiru T. Utilization of non-skilled birth attendants in Northern Nigeria: a rough terrain to the health-related MDGs. African Journal of Reproductive Health. 2010;14(2):37-45.

19. Ogbo FA, Trinh FF, Ahmed KY, Senanayake P, Rwabilimbo AG, Uwaibi NE, et al. Prevalence, Trends, and Drivers of the Utilization of Unskilled Birth Attendants during Democratic Governance in Nigeria from 1999 to 2018. International journal of environmental research and public health. 2020;17(1):372.

20. World Health Organization. Maternal Mortality: Key Facts Geneva, Switzerland: WHO; 2021 [Cited on 29 September 2021]. Available from: https://www.who.int/news-room/fact-sheets/detail/maternal-mortality.

21. World Health Organization Department of Reproductive Health and Research. Making pregnancy safer: the critical role of the skilled attendant Geneva, Switzerland: WHO, ICM and FIGO

2021 [Cited on 27 September 2021]. Available from: https://www.who.int/reproductivehealth/publications/maternal_perinatal_health/9241591692/en/.

22. Namusonge LN, Kabue P, Sharma RR. Women s persistent utilisation of unskilled birth attendants in Kakamega County, Kenya. East African Medical Journal. 2016;93:691-711.

23. Choguya NZ. Traditional and Skilled Birth Attendants in Zimbabwe: A Situational Analysis and Some Policy Considerations. Journal of Anthropology. 2015;2015:215909.

24. Tabong PT-N, Kyilleh JM, Amoah WW. Reasons for the utilization of the services of traditional birth attendants during childbirth: A qualitative study in Northern Ghana. Women's Health. 2021;17:17455065211002483.

25. Ribeiro Sarmento D. Traditional Birth Attendance (TBA) in a health system: what are the roles, benefits and challenges: A case study of incorporated TBA in Timor-Leste. Asia Pacific Family Medicine. 2014;13(1):12.

List of Figures

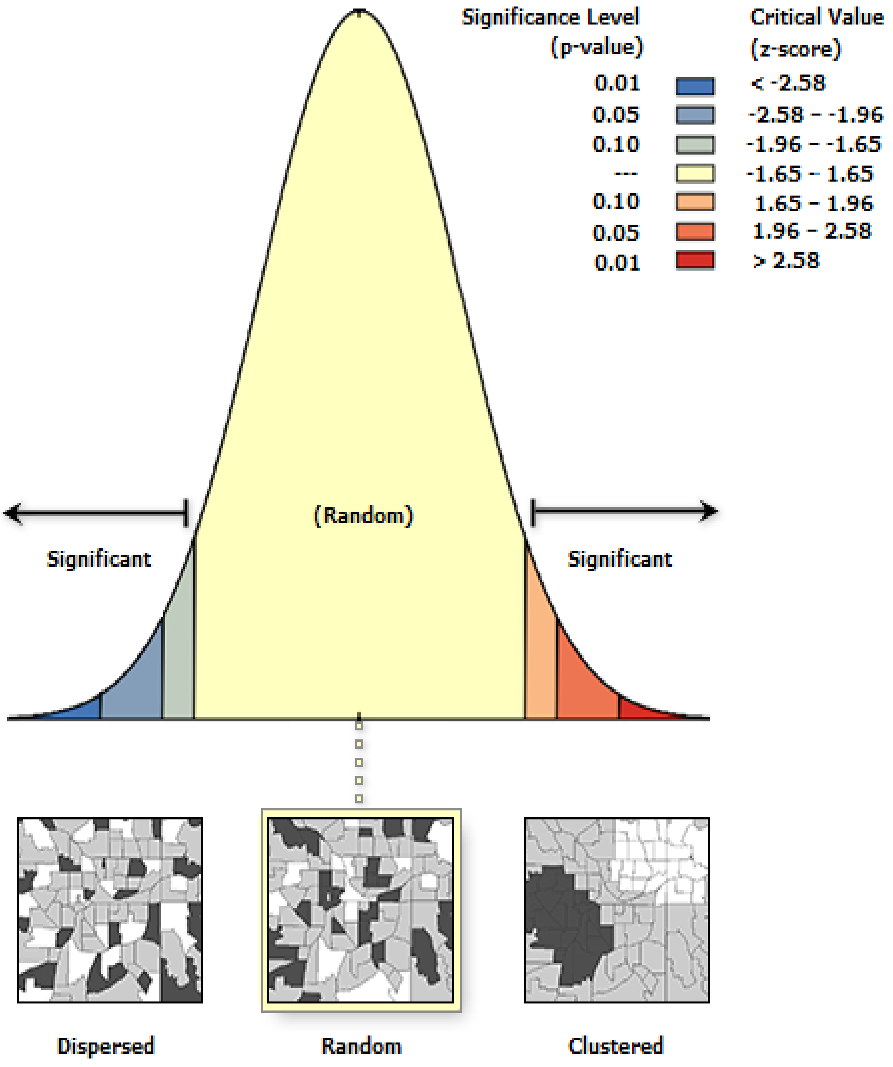

Figure 1: Spatial Autocorrelation analysis

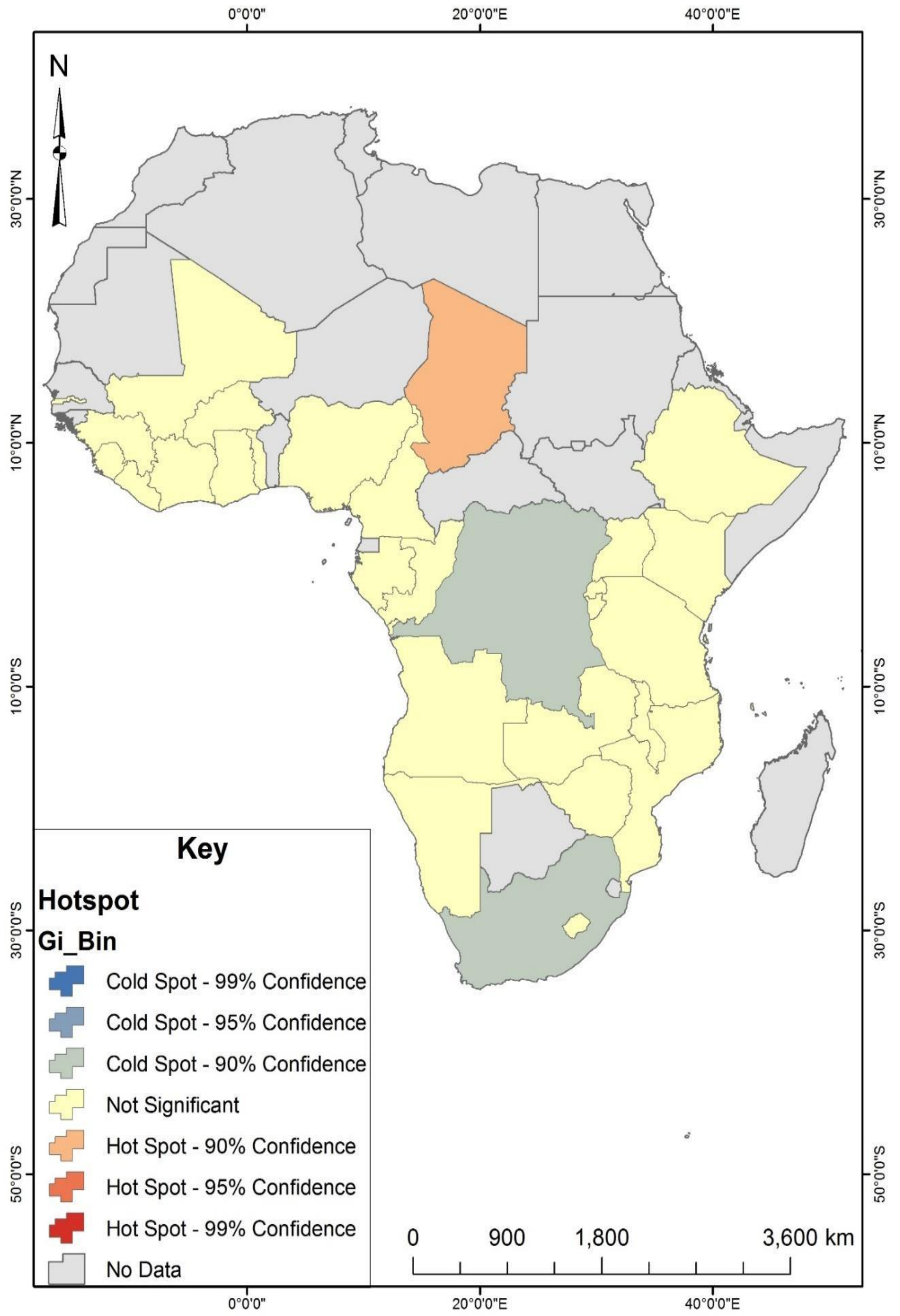

Figure 2: Hotspot analysis

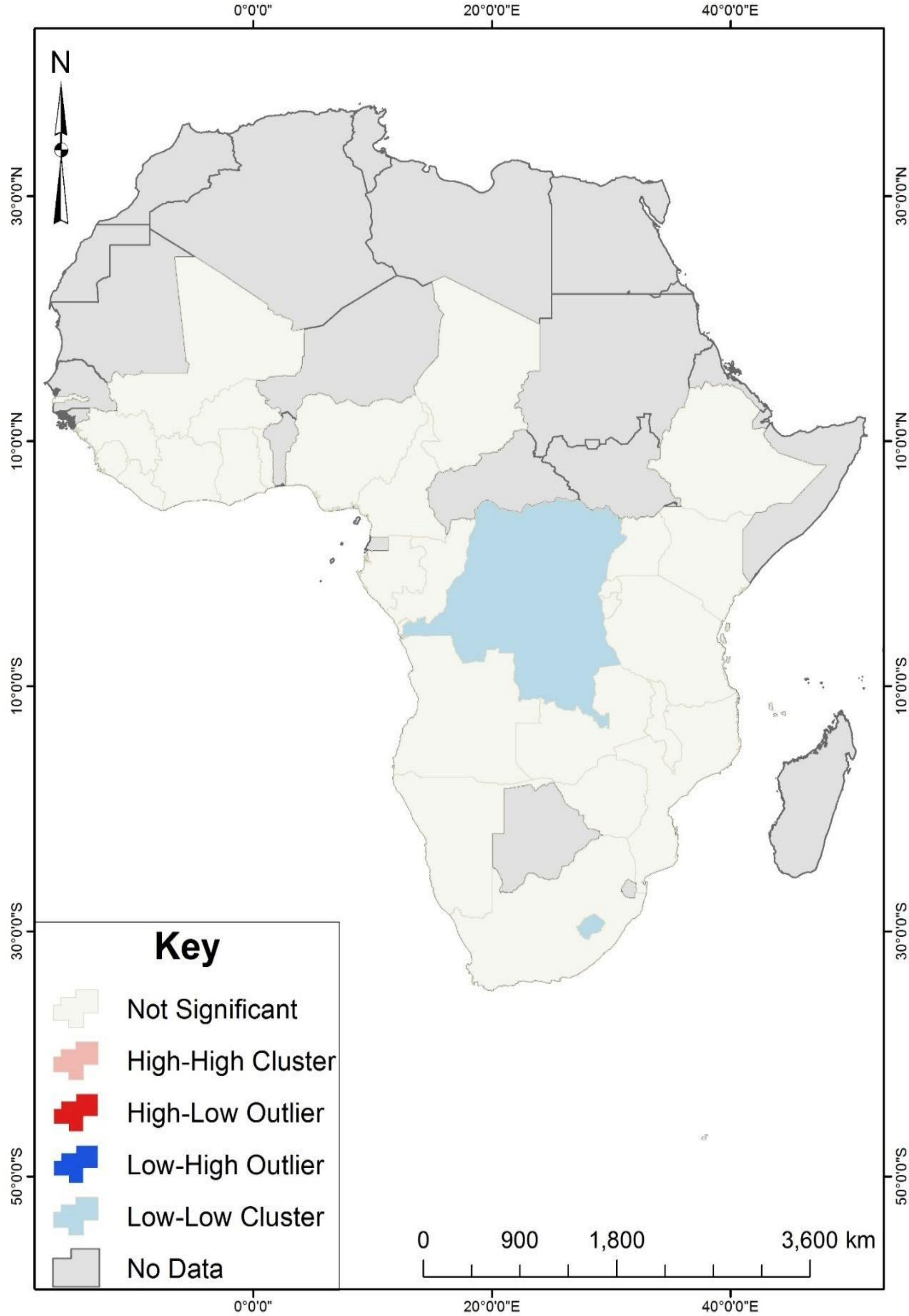

Figure 3: Cluster and Outlier analysis

Table 1: Proportion of unskilled birth assistance in Sub-Saharan Africa

Country		Frequency	Percentage	Proportion of unskilled birth assistance
1.	Angola 2015-2016 AO	8,577	4.3	50.0
2.	Burkina Faso 2010 BF	10,255	5.1	26.2
3.	Burundi 2016-2017 BU	16,316	8.1	7.9
4.	Cameroon 2018 CM	6,288	3.1	26.4
5.	Chad 2014-2015 TD	3,652	1.8	68.9
6.	Comoros 2012 KM	1,824	0.9	15.1
7.	Congo 2011-2012 CG	6,305	3.1	12.0
8.	Congo DR 2013-2014 CD	10,963	5.5	20.4
9.	Cote d'Ivoire 2011-2012 CI	5,304	2.6	40.8
10.	Ethiopia 2016 ET	7,052	3.5	60.4
11.	Gabon 2012 GA	3,986	2.0	15.9
12.	Gambia 2013 GM	5,677	2.8	16.8
13.	Ghana 2014 GH	4,201	2.1	25.9
14.	Guinea 2018 GN	5,362	2.7	44.2
15.	Kenya 2014 KE	6,896	3.4	40.1
16.	Lesotho 2014-2015 LS	995	0.5	18.5
17.	Liberia 2013 LB	4,186	2.1	16.0
18.	Malawi 2015-2016 MW	12,544	6.3	7.4
19.	Mali 2018 ML	6,157	3.1	29.5
20.	Mozambique 2011 MZ	7,349	3.7	36.2
21.	Namibia 2013 NM	3,802	1.9	11.8
22.	Nigeria 2018 NG	15,091	7.5	46.1
23.	Rwanda 2014-2015 RW	5,796	2.9	7.5
24.	Sierra Leone 2019 SL	7,257	3.6	12.5
25.	South Africa 2016 ZA	1,438	0.7	3.3
26.	Tanzania 2015-2016 TZ	6,822	3.4	32.8
27.	Togo 2013-2014 TG	4,942	2.5	44.6
28.	Uganda 2016 UG	9,844	4.9	22.9
29.	Zambia 2013-2014 ZM	1,438	3.6	18.3
30.	Zimbabwe 2015 ZW	4,667	2.3	15.3
	All Countries	200,736	100	26.7

Table 2: Socio-demographic characteristics and unskilled birth assistance

Variables	Frequency (n=200,736)	Percentage	Proportion of unskilled birth assistance
Place of residence			
Urban	63,953	31.9	12.1
Rural	136,783	68.1	33.6
Wealth status			
Poorest	48,435	24.1	43.7
Poorer	42,777	21.3	33.9
Middle	39,756	19.8	26.2
Richer	36,506	18.2	15.6
Richest	33,262	16.6	5.7
Age			
Less than 20	15,121	7.5	25.2
20 – 29	96,268	48.0	25.1
30 – 49	89,347	44.5	28.8
Level of education			
No education	69,532	34.7	43.3
Primary	72,093	35.9	24.5
Secondary	52,465	26.1	10.9
Higher	6,646	3.3	3.0
Marital status			
Never in union	17,100	8.5	16.3
Married	131,305	65.4	29.1
Cohabitation	36,431	18.1	24.1
Widowed	3,141	1.6	31.9
Divorced	3,591	1.8	26.4
Separated	9,168	4.6	21.1
Occupation			
Not working	52,906	26.4	27.4
Working	147,830	73.6	26.5
**Age at first birth**			
Less than 20	118,463	59.0	30.5
20 – 29	78,561	39.1	21.6
30 – 49	3,712	1.9	16.2
Birth order			
1	42,414	21.1	16.4
2 – 3	70,022	34.9	23.4
4 or more	88,300	44.0	34.3
Mass media exposure			
No	78,388	39.1	36.7
Yes	122,348	60.9	20.4
Getting medical help for self: permission to go			
Big problem	37,391	18.6	35.7
Not a big problem	163,345	81.4	24.7

Getting medical help for self: getting money needed for treatment			
Big problem	111,763	55.7	31.7
Not a big problem	88,973	44.3	20.6
Getting medical help for self: distance to health facility			
Big problem	82,583	41.1	36.5
Not a big problem	118,153	58.9	19.9
Desire for birth			
Then	129,671	64.6	27.3
Later	12,237	6.1	31.8
No more	58,828	29.3	24.5
Antenatal care visits			
Less than 4	85,250	42.5	40.5
4 or more	115,486	57.5	16.6
Under-five mortality			
No	192,492	95.9	26.5
Yes	8,244	4.1	32.3

Table 3: Logistic regression of factors associated with unskilled birth attendance

Variables	Model 1<br>Odds Ratio<br>(95% Confidence interval)	Model 2<br>Adjusted Odds Ratio<br>(95% Confidence interval)
Place of residence		
Urban	0.27***(0.26, 0.28)	0.53***(0.51, 0.55)
Rural	Ref	Ref
Wealth status		
Poorest	Ref	Ref
Poorer	0.66***(0.64, 0.68)	0.73***(0.70, 0.75)
Middle	0.46***(0.44, 0.47)	0.57***(0.55, 0.59)
Richer	0.24***(0.23, 0.25)	0.40***(0.38, 0.41)
Richest	0.07***(0.07, 0.08)	0.23***(0.22, 0.24)
Age		
Less than 20	1.01(0.97, 1.05)	1.11***(1.05, 1.17)
20 – 29	Ref	Ref
30 – 49	1.21***(1.18, 1.23)	0.92***(0.89, 0.95)
Level of education		
No education	2.36***(2.31, 2.41)	1.57***(1.52, 1.62)
Primary	Ref	Ref
Secondary	0.38***(0.37, 0.39)	0.58***(0.56, 0.61)
Higher	0.10***(0.08, 0.11)	0.26***(0.22, 0.30)
Marital status		
Never in union	0.47***(0.45, 0.49)	0.97(0.92, 1.03)
Married	Ref	Ref
Cohabitation	0.77***(0.75, 0.79)	0.95**(0.91, 0.98)
Widowed	1.14**(1.06, 1.23)	1.14**(1.04, 1.25)
Divorced	0.87***(0.81, 0.94)	1.11*(1.01, 1.22)
Separated	0.65***(0.62, 0.68)	0.95(0.89, 1.01)
Occupation		
Not working	1.04***(1.02	1.03*(1.00, 1.07)
Working	Ref	Ref
**Age at first birth**		
Less than 20	Ref	Ref
20 – 29	0.63***(0.62, 0.64)	0.90***(0.87, 0.92)
30 – 49	0.44***(0.40, 0.48)	0.81***(0.72, 0.91)
Birth order		
1	0.38***(0.36, 0.39)	0.51***(0.49, 0.54)
2 – 3	0.59***(0.57, 0.60)	0.79***(0.76, 0.82)
4 or more	Ref	Ref
Mass media exposure		
No	2.26***(2.21, 2.30)	1.25***(1.22, 1.29)
Yes	Ref	Ref
Getting medical help for self: permission to go		
Big problem	1.69***(1.65, 1.73)	1.02(0.98, 1.05)
Not a big problem	Ref	Ref
Getting medical help for self: getting money needed for treatment		
Big problem	Ref	Ref
Not a big problem	0.56***(0.55, 0.57)	0.95***(0.92, 0.98)

Getting medical help for self: distance to health facility		
Big problem	2.31***(2.26, 2.35)	1.49***(1.45, 1.53)
Not a big problem	Ref	Ref
Desire for birth		
Then	Ref	Ref
Later	1.24***(1.19, 1.29)	0.96(0.91, 1.01)
No more	0.86***(0.85, 0.88)	0.86***(0.83, 0.89)
Antenatal care visits		
Less than 4	3.41***(3.34, 3.48)	2.61***(2.54, 2.68)
4 or more	Ref	Ref
Under five mortality		
No	Ref	Ref
Yes	1.32***(1.26, 1.39)	1.10***(1.04, 1.17)
Country		
Angola 2015-2016 AO	11.73***(10.92, 12.59)	23.22***(21.34, 25.26)
Burkina Faso 2010 BF	3.98***(3.70, 4.28)	3.09***(2.86, 3.34)
Burundi 2016-2017 BU	Ref	Ref
Cameroon 2018 CM	4.19***(3.87, 4.54)	7.36***(6.72, 8.06)
Chad 2014-2015 TD	25.93***(23.69, 28.38)	24.09***(21.72, 26.71)
Comoros 2012 KM	2.08***(1.81, 2.39)	2.60***(2.22, 3.03)
Congo 2011-2012 CG	1.60***(1.46, 1.76)	2.12****1.91, 2.35)
Congo DR 2013-2014 CD	2.99***(2.78, 3.22)	3.12***(2.88, 3.38)
Cote d'voire 2011-2012 CI	8.07***(7.46, 8.73)	8.49***(7.77, 9.27)
Ethiopia 2016 ET	17.82***(16.54, 19.20)	19.49***(17.87, 21.25)
Gabon 2012 GA	2.21***(2.00, 2.45)	3.75***(3.34, 4.21)
Gambia 2013 GM	2.37***(2.17, 2.59)	3.73***(3.38, 4.12)
Ghana 2014 GH	4.38***(4.00, 4.78)	9.94***(8.99, 10.99)
Guinea 2018 GN	9.26***(8.56, 10.01)	8.63***(7.90, 9.43)
Kenya 2014 KE	7.85***(7.28, 8.45)	14.69***(13.50, 16.00)
Lesotho 2014-2015 LS	2.66***(2.24, 3.15)	9.30***(7.70, 11.24)
Liberia 2013 LB	2.22***(2.01, 2.46)	3.39***(3.04, 3.78)
Malawi 2015-2016 MW	0.94(0.86, 1.02)	1.04(0.95, 1.14)
Mali 2018 ML	4.89***(4.52, 5.29)	4.90***(4.49, 5.36)
Mozambique 2011 MZ	6.65***(6.17, 7.16)	10.63***(9.77, 11.57)
Namibia 2013 NM	1.56***(1.40, 1.75)	4.62***(4.07, 5.25)
Nigeria 2018 NG	10.00***(9.37, 10.68)	22.32***(20.72, 24.05)
Rwanda 2014-2015 RW	0.95(0.85, 1.07)	1.34***(1.19, 1.51)
Sierra Leone 2019 SL	1.68***(1.53, .83)	2.30***(2.09, 2.53)
South Africa 2016 ZA	0.40***(0.30, 0.54)	1.54**(1.14, 2.09)
Tanzania 2015-2016 TZ	5.70***(5.29, 6.16)	8.57***(7.88, 9.33)
Togo 2013-2014 TG	9.44***(8.71, 10.23)	13.40***(12.25, 14.66)
Uganda 2016 UG	3.48***(3.23, 3.75)	5.26***(4.85, 5.70)
Zambia 2013-2014 ZM	2.62***(2.42, 2.85)	4.14***(3.78, 4.54)
Zimbabwe 2015 ZW	2.11***(1.91, 2.33)	5.92***(5.30, 6.60)

*P<0.05, **p<0.01, ***p<0.001; Ref=Reference category; Mean vif= 1.61
