# Supplementary material for: Factors associated with unskilled birth attendance among women in sub-Saharan Africa: A multivariate-geospatial analysis of demographic and health surveys
Source: PLoS One. 2023 Feb 2;18(2):e0280992. doi: 10.1371/journal.pone.0280992 (PMC9894461; doi:10.1371/journal.pone.0280992)
Supplement: Attachment — Submitted filename: PONE-D-21-36273 #Comments and suggestions.docx [file pone.0280992.s001.docx]

**Comments and suggestions**

Line-38: “older current age and age at birth”

- The association direction for age at birth is not specified.
- Does the word “older” applies for both or not? If it applies for both then better if you write separately as older current age, older age at birth …

Line 38 and 39:

- The two variables; rich wealth status Vs not having money problems for healthcare
- Both are complementary to each other. So, I recommend to select and use one. Wealth status is better.

Line 43:

- Why Burundi?
- Lowest proportion is from South Africa (SA) (less exposed to the outcome; meaning less proportion of the outcome is indicated from the descriptive analysis. So, I recommend to make SA as reference)
- Then what is the implication behind the result? All counties have higher odds?

Line 78-79:

- Add something about the spatial pattern of the issue in addition to what you stated.

Line 93:

- The word “Explored” is not the appropriate action verb for such type of quantitative analysis. Better to use “identified” or “examined” …

Line 95:

- The same comment as line 93 (Exploring???)

Line 101:

- Could it be different for countries? If it the year is different how you handle the analysis? How do you control the effect of time? So, it is good if you make DHS data sets which are produced in the same year across different countries. Otherwise, the result is biased due to time effect or make it time series analysis?

Line 180-187:

- The description is not as such important as far as these variables are included in the regression model. The proportion (percentage) here is crude and it doesn’t add any value. So, delete it and try to include all variables in to the model and report their adjusted effect on the outcome.

Line 194:

- Make is showed i.e.) add “ed”

Line 202:

- Delete (Table 3).; as it is reported at the end of the paragraph.

Line 213:

- Delete the word “see” as it is not important (be consistent).

Line 222:

- A dd (Table 3) immediately after the word child.

Line 254-258:

- The paragraph you stated as a starting point and discussion is not directly addressed through this analysis. So, I recommend to write based on the study objectives. This study didn’t address the effect of unskilled birth attendance on maternal death though it is addressed by other studies. So, try to focus on the study objectives. This study has nothing related to this issue.
- Start with your main finding and then try to compare to others studies and policy implications
- The proportion of the outcome is not discussed well. so, discuss the percentage in relation to expected proportion based on global initiatives

Line 263-265

- Delete it; what is the important? Or make it short (paraphrase it).

Line 266-280:

- It is good you have stated some important statement for discussion. But these significant variables must be discussed separately with their corresponding justification. E.g.) why younger women higher odds of utilizing the service? Labor and child birth experience Vs Woman’s age and then Vs unskilled birth attendance?
- So, the same is true for other variables found to be significant in the final model need to be discussed.

Line 283:

- The use of conjunction (“Interestingly,”) is not correct for under five demise Vs Unskilled birth attendance.
- The reasoning stated in the document is not convincing. So, try to make it informative and convincing? Imogie (it is single study) and it is difficult to compare this study where it is the analysis many countries with a single study in Nigeria?

Line 281-296:

- The reasoning or justification need to be modified.

Line 297-307:

- What could be the reason for hotspot for some countries and coldspot for some countries? Justification if you can?

Line 309-316: regarding the strength

- Statements about strength of the study need to be paraphrased. Please come to the point in short (the only strength could be using large sample size; nothing else). Using logistic model can not be a strength. The spatial can be a strength.

Line 316-327:

- Please try to state your own study or analysis limitation.
- And then what are the potential limitation that you will share with DHS protocol.
- Being cross-sectional can’t be a limitation for your analysis. (Causal analysis is not your plan/objective)
- Still this subsection needs to paraphrased.

Line 357: delete the “.” Before the reference

- The points stated under “Implications for policy and practice” can be written under discussion and conclusion based on the variables you discussed. Under discussion you can finalize with its public health policy and implementation implications
- Similar topic is done in Ghana by similar author; so, why you include Ghana in this analysis? What makes different from that study? What is new?
- The key legends for figure 2 and 3 Vs the colours in the figure are not comparable and this hide the information that can be captured from the figures.
